# Sampling and diversity of *Escherichia coli* from the enteric microbiota in patients with *Escherichia coli* bacteraemia

**DOI:** 10.1186/s13104-019-4369-y

**Published:** 2019-06-13

**Authors:** Mia Mosavie, Oliver Blandy, Elita Jauneikaite, Isabel Caldas, Matthew J. Ellington, Neil Woodford, Shiranee Sriskandan

**Affiliations:** 1 Health Protection Research Unit in Healthcare Associated Infections and Antimicrobial Resistance, Department of Medicine, Imperial College London, Hammersmith Campus, Du Cane Road, London, W12 0NN UK; 2 Antimicrobial Resistance and Healthcare Associated Infections (AMRHAI) Reference Unit, National Infection Service, Public Health England, London, UK

**Keywords:** *Escherichia coli*, Microbiota, RAPD, Diversity, Bacteraemia, Stool, Rectal swab, Sepsis

## Abstract

**Objective:**

The increase in *Escherichia coli* bloodstream infections mandates better characterisation of the relationship between commensal and invasive isolates. This study adopted a simple approach to characterize *E. coli* in the gut reservoir from patients with either *E. coli* or other Gram-negative bacteraemia, or those without bacteraemia, establishing strain collections suitable for genomic investigation. Enteric samples from patients in the three groups were cultured on selective chromogenic agar. Genetic diversity of prevailing *E. coli* strains in gut microbiota was estimated by RAPD-PCR.

**Results:**

Enteric samples from *E. coli* bacteraemia patients yielded a median of one *E. coli* RAPD pattern (range 1–4) compared with two (range 1–5) from groups without *E. coli* bacteraemia. Of relevance to large-scale clinical studies, observed diversity of *E. coli* among hospitalised patients was not altered by sample type (rectal swab or stool), nor by increasing the colonies tested from 10 to 20. Hospitalised patients demonstrated an apparently limited diversity of *E. coli* in the enteric microbiota and this was further reduced in those with *E. coli* bacteraemia. The reduced diversity of *E. coli* within the gut during *E. coli* bacteraemia raises the possibility that dominant strains may outcompete other lineages in patients with bloodstream infection.

**Electronic supplementary material:**

The online version of this article (10.1186/s13104-019-4369-y) contains supplementary material, which is available to authorized users.

## Introduction

The increasing burden of *Escherichia coli* bacteraemia in the United Kingdom and dominance of antimicrobial-resistant lineages worldwide [1–4] point to a need for better understanding of the reservoir and diversity of *E. coli* in the gut microbiota of those affected by invasive *E. coli* infections. *E. coli* is present in abundance in the adult gut microbiota, however the diversity of *E. coli* strains within the gut reservoir has only been studied in limited groups that do not include bacteraemia patients [5–10].

The enteric microbiota of such patients are hard to study, as bacteraemic patients will be acutely unwell, and stool samples are usually not readily available or clinically indicated, albeit that routinely-collected samples could represent an accessible sample source. Patients with suspected bacteraemia will usually receive empirical antimicrobial therapy within an hour of admission to hospital, consistent with international guidance on sepsis [11], thus all enteric samples are potentially antibiotic-exposed and may also be refrigerated for long periods of time before reaching the diagnostic laboratory, in turn potentially affecting microbiota [12].

Our aims were to establish methods to adequately sample *E. coli* strains present within the intestinal microbiota of hospital patients with *E. coli* bacteraemia; to evaluate control hospitalised groups; to assess scalability; and establish a sample collection for future large-scale genomic studies. Samples would need to be representative of the prevailing *E. coli* strains present in each patient without necessarily providing exhaustive insight into rare variants. The ability to detect *E. coli* variants in the enteric microbiota is related only to the underlying prevalence of such variants and how many colonies are counted [13]; to have a 90% likelihood of detecting a strain present in 20% of colonies, one needs to sample 11 colonies, while for a strain so rare it is present in just 5% of colonies, one needs to sample 45 colonies. Randomly Amplified Polymorphic DNA (RAPD) analysis can provide a relatively crude, but cheap, molecular method to differentiate strains based on amplicon banding patterns [8, 14, 15] and was used to inform sampling strategy and characterise the samples obtained.

## Main text

### Methods

#### Patients and samples

Enteric samples were stool or rectal swabs from hospital in-patients obtained within 72 h of onset of Gram negative bacteraemia in west London National Health Service (NHS) Teaching Hospital sites between 1st July 2015 and 4th August 2016. Enteric samples were sought from patients prior to final microbiological identification thus were exposed to the same antimicrobial agents; groups were subsequently categorised into confirmed *E. coli* bacteraemia or other Gram negative non-*E. coli* bacteraemia. Controls were selected from inpatients within the same hospital wards that had not received an antibiotic in the preceding 30 days. Samples were obtained with informed consent except where rectal swabs had already been submitted to the hospital diagnostic laboratory for routine screening. In total, the study included 462 enteric samples (Additional file 1: Figure S1). Samples were refrigerated and analysed within 48 h unless otherwise stated, after any routine diagnostic testing had been completed. Preliminary studies demonstrated that refrigeration in the research lab for 48–72 h had little, or no, effect on the retrieval of *E. coli* from enteric samples though yield reduced by 20–25% over 10 days (data not shown). The study was approved by the Camden and Islington National Research Ethics Committee (Reference 14/LO/2217).

#### Culture methods

Approximately 10–60 mg of stool was suspended in 3 ml 0.9% saline to achieve an optical density of A_600_ 0.1 [16]. The suspension was diluted 100-fold in 0.9% saline and 100 µl (~ 300–500 colony forming units, CFU) cultured on coliform-selective chromogenic agar (Brilliance™ *E. coli* coliform Selective Agar, Oxoid, UK) and Columbia Blood Agar (CBA) (EO Labs, UK) at 37 °C overnight in 5% CO_2_. Samples with low counts (0–5 CFU) after 24 h growth were re-plated using 100 µl of undiluted suspension. Rectal swabs were plated directly on agar as described above. To confirm presence of *E. coli* in a selection of stool samples, DNA was extracted directly from stool using Qiagen QIAamp DNA Stool Extraction Kit (Qiagen, UK) and *gadA* polymerase chain reaction (PCR) was performed as previously reported [17] (Additional file 1: Table S1). Samples were separately plated and all colonies frozen in glycerol bead stocks for future use.

#### Sampling strategy

To determine the optimum number of colonies to pick from patient samples, groups of 5, 10, 15, and 20 *E. coli* single colonies were selected from 10 randomly selected samples. Donors were *E. coli* bacteraemia patients receiving antibiotics (n = 5), and hospitalised patients who did not have bacteraemia and were not receiving antibiotics (n = 5). Individual colonies were boiled at 90 °C for 10 min to lyse the cells and release DNA; these were then subject to randomly-amplified-polymorphic-DNA (RAPD)-PCR using primers previously reported [18] (Additional file 1: Table S1). The number of different RAPD patterns obtained from 5, 10, 15 or 20 single *E. coli* colonies was compared for each sample.

#### Comparison of *E. coli* diversity between patient groups

Single purple colonies from chromogenic agar identified as *E. coli* were randomly selected from different quadrants of the chromogenic agar plate, and sub-cultured on to LB agar (Oxoid, UK). Ten colonies per sample were subject to DNA extraction and RAPD-PCR as described above. The number of different RAPD patterns obtained was compared for each of the patient groups. Groups were compared pairwise by Mann–Whitney test; groups of more than two were compared by Kruskal–Wallis (Graph Pad Prism).

### Results and discussion

#### Sampling strategy

Although up to 10 distinct *E. coli* lineages can be present in the normal human enteric microbiota, medians of 2–3 types have been reported in patients in the community [6–10]. For a large clinical study in bacteraemia patients, that might include 100–200 hospitalised cases, exhaustive molecular analysis of 1000’s of *E. coli* colonies would not be feasible. To determine a suitable number of *E. coli* colonies to analyse per patient when evaluating the diversity of *E. coli* lineages in the gut microbiota of hospitalized patients, we compared RAPD patterns of 20, 15, 10 and 5 *E. coli* colonies cultured from ten different in-patient faecal samples. We included equal numbers of samples from those diagnosed with bacteraemia and from those who had not been receiving any antibiotics for 30 days prior to sample collection.

**Fig. 1:**
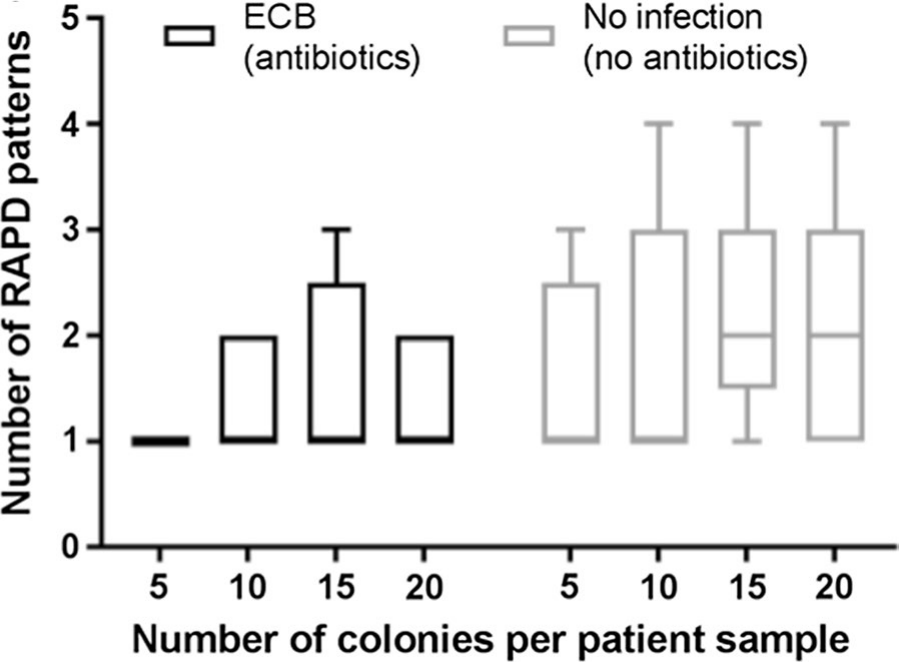
Number of RAPD patterns detected when testing 5, 10, 15 or 20 *E. coli* colonies per patient sample. Enteric samples were tested from *E. coli* bacteraemia (ECB) patients (n = 5, receiving antibiotics), and non-antibiotic-exposed inpatient controls (n = 5, no infection, not receiving antibiotics). Different numbers of colonies were evaluated, as indicated and the number of distinct RAPD patterns enumerated. Plot shows median and interquartile range

Examination of 15 colonies allowed detection of increased diversity in just one sample tested in each group compared with examination of 10 colonies. Further expansion to examine 20 colonies did not increase the observed diversity of *E. coli* per sample in either group. Taking all the data together, there was no difference in the number of potential different genotypes detectable by RAPD-PCR when selecting 10 or 20 colonies (p > 0.05) (Fig. 1 and Additional file 1: Table S2). As such, for subsequent work, a practical approach of selecting 10 colonies per patient was adopted.

#### Population of *E. coli* in different patient groups

Enteric samples were collected from a larger cohort of 74 *E. coli* bacteraemia patients (receiving empiric antibiotics) and 70 control in-patients not receiving antibiotics. To control for the potential effect of antibiotic exposure, we included an additional group of 42 other Gram-negative (non *E. coli*) bacteraemia patients, who received the same empiric antibiotics as the *E. coli* bacteremia group. Samples were sought from all eligible patients during the study period and RAPD-PCR was undertaken in all cases where enteric samples yielded at least 10 colonies of viable *E. coli* on subculture (Additional file 1: Figure S1).

**Fig. 2:**
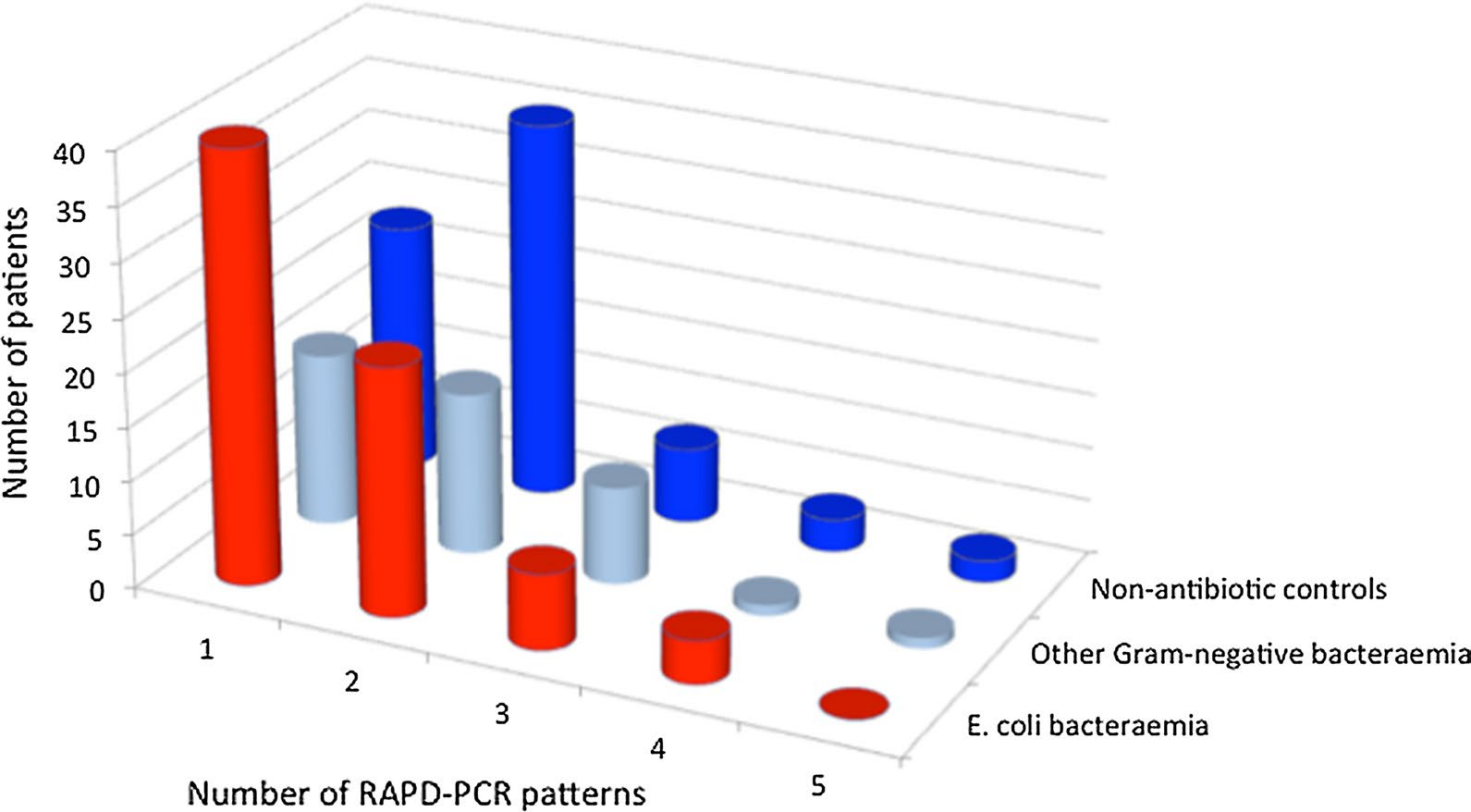
Diversity of *E. coli* in enteric microbiota from patients with *E. coli* bacteraemia compared with other patient groups. Frequency of different numbers of RAPD patterns among *E. coli* bacteraemia patients (n = 74), patients with other Gram negative bacteraemia (n = 42), and non-antibiotic exposed inpatient controls who had no infection and were not taking antibiotics (n = 70). Although a difference was detected between the two main groups (*E. coli* bacteraemia and non-antibiotic inpatient controls, p = 0.029), there were no overall differences between the three groups (p = 0.06)

The median number of *E. coli* RAPD patterns recovered from 70 hospital in-patient controls, who were not receiving antibiotics, was two (range 1–5), in contrast to the 74 *E. coli* bacteraemia patients in whom a median of one RAPD type (range 1–4) was detected (p = 0.029). Despite being similarly antibiotic-treated, the median number of RAPD patterns detected in the 42 non-*E. coli* Gram negative bacteraemia samples analysed was two (range 1–5) although the differences between the three groups overall was not significant (p = 0.06) (Fig. 2).

We considered whether the type of sample affected our results and so compared data obtained from rectal swabs and faecal samples; rectal swabs were performed largely for the purpose of screening for carriage of carbapenem-resistant organisms using risk-based algorithms and could represent a marker of prior healthcare exposure. Importantly, however, there was no significant difference between the two types of sample (Additional file 1: Figure S2). Rectal swabs may be obtained earlier during each hospital admission than stool samples, reducing the effect of recent antibiotic exposure on the diversity detected. The value of using rectal swabs is a useful observation, since inpatients are often unable to provide a faecal sample, while rectal swabs are increasingly obtained as part of routine screening practice.

Overall, we found a narrower diversity of *E. coli* in *E. coli* bacteraemia patients compared to control hospitalised patients. Although our study was not sufficiently powered to directly compare the *E. coli* bacteraemia group with the ‘other Gram negative bacteraemia’ group, our data suggest that the reduced diversity of *E. coli* in the enteric microbiota of *E. coli* bacteraemia patients may be specific to this group, and not necessarily related to concurrent antimicrobial exposure. We speculate that pathogenic *E. coli* strains causing bacteraemia may outcompete and dominate the gut microbiota. Whether this is related to past antimicrobial consumption or other pressures affecting the enteric reservoir of in-patients will require further study using specifically selected populations of patients with known antimicrobial history.

## Limitations

Our study had limitations, in that enteric samples were only available from around a quarter of eligible patients with bacteraemia, and, for practical reasons, efforts to culture *E. coli* from all patients did not include heat shock or novobiocin-enrichment which, in separate studies, we found did improve yield (not shown). However, the number of samples evaluated was high, and there is no evidence to suggest these limitations would have affected any one patient group more than another. The number of different *E. coli* lineages detected in the enteric microbiota of our control groups is similar to that reported by other investigators [8] although, to our knowledge, acutely unwell hospital inpatients have not been studied previously.

A practical decision was made to sample ten colonies per patient, a strategy that means uncommon variants (present at a level of 10% or less) in the enteric sample will be identified less frequently (~ 35%) [13]. In our pilot study of hospitalised patients, increasing sampling from 10 to 20 colonies did not increase the yield of genetically distinct *E. coli* types. Notably this part of the study was markedly under-powered to detect very small differences such as those observed, and would have required many 100’s of samples to detect a clear difference; increasing colony sampling may have a greater effect in alternate non-hospitalised or healthy patient populations.

RAPD-PCR can reliably investigate the diversity of *E. coli* subclones in enteric microbiota [15] although cannot be used to finely discriminate between isolates at the genomic level; results may be refined using genomic DNA extraction methods. It does however provide an affordable and scalable method to determine the number of isolates to study using more expensive methods such as genome sequencing.

## Additional file

**Additional file 1.** Sampling and diversity of *Escherichia coli* from the enteric microbiota in patients with *Escherichia coli* bacteraemia. **Table S1.** Primers used in this study. **Table S2.** Frequency of different RAPD patterns in each patient sample. **Figure S1.** Sample recruitment for study. **Figure S2.** *E. coli* diversity in rectal swab and stool samples.

## Data Availability

All data generated or analysed during this study are included in this published article (and its additional files).

## Abbreviations

RAPD: randomly amplified polymorphic DNA
PCR: polymerase chain reaction
CFU: colony forming units
CBA: Columbia Blood Agar
NHS: National Health Service
NIHR HPRU: National Institute for Health Research Health Protection Research Unit
